# Wearable Sensors and Artificial Intelligence for the Diagnosis of Parkinson’s Disease

**DOI:** 10.3390/jcm14124207

**Published:** 2025-06-13

**Authors:** Yacine Benyoucef, Islem Melliti, Jouhayna Harmouch, Borhan Asadi, Antonio Del Mastro, Diego Lapuente-Hernández, Pablo Herrero

**Affiliations:** 1 SPACEMEDEX, 930 Rte des Dolines, Valbonne Sophia-Antipolis, 06560 Valbonne, France; mila.melliti@gmail.com (I.M.);; 2 Department of Physiatry and Nursing, Faculty of Health Sciences, University of Zaragoza, 50009 Zaragoza, Spain; basadi@iisaragon.es (B.A.); d.lapuente@unizar.es (D.L.-H.); pherrero@unizar.es (P.H.); 3 iHealthy Research Group, University of Zaragoza/IIS Aragon, 50009 Zaragoza, Spain; 4 ALDEBRAN, Via Dalmine 10/A, 24035 Curno, BG, Italy; info@aldebran.com

**Keywords:** Parkinson’s disease, human activity recognition, wearable sensors, artificial intelligence, machine learning, rehabilitation

## Abstract

**Background/Objectives:** This study explores the integration of wearable sensors and artificial intelligence (AI) for Human Activity Recognition (HAR) in the diagnosis and rehabilitation of Parkinson’s disease (PD). The objective was to develop a proof-of-concept model based on internal reproducibility, without external generalization, that is capable of distinguishing pathological movements from healthy ones while ensuring clinical relevance and patient safety. **Methods**: Nine subjects, including eight patients with Parkinson’s disease and one healthy control, were included. Motion data were collected using the Motigravity platform, which integrates inertial sensors in a controlled environment. The signals were automatically segmented into fixed-length windows, with poor-quality segments excluded through preprocessing. A hybrid CNN-LSTM (Convolutional Neural Networks—Long Short-Term Memory) model was trained to classify motion patterns, leveraging convolutional layers for spatial feature extraction and LSTM layers for temporal dependencies. The Motigravity system provided a controlled hypogravity environment for data collection and rehabilitation exercises. **Results**: The proposed CNN-LSTM model achieved a validation accuracy of 100%, demonstrating classification potential. The Motigravity system contributed to improved data reliability and ensured patient safety. Despite increasing class imbalance in extended experiments, the model consistently maintained perfect accuracy, suggesting strong generalizability after external validation to overcome the limitations. **Conclusions**: Integrating AI and wearable sensors has significant potential to improve the HAR-based classification of movement impairments and guide rehabilitation strategies in PD. While challenges such as dataset size remain, expanding real-world validation and enhancing automated segmentation could further improve clinical impact. Future research should explore larger cohorts, extend the model to other neurodegenerative diseases, and evaluate its integration into clinical rehabilitation workflows.

## 1. Introduction

Parkinson’s disease (PD) is the second most prevalent neurodegenerative disorder, affecting over 10 million individuals worldwide [1]. Traditional clinical diagnosis primarily relies on subjective assessments, which can often lead to delayed intervention. Recent advancements in Human Activity Recognition (HAR), facilitated by wearable motion sensors and machine learning techniques, have demonstrated outstanding potential for detecting early motor impairments [2,3,4]. However, real-world applications for diagnostic purposes remain limited, especially in early-stage PD, where clinical signs may be subtle and fluctuating.

HAR enables a detailed and precise analysis of human movement by integrating motion capture technologies, computational techniques, and statistical models. This interdisciplinary field has significant applications in medicine, well-being, sports science, and behavioral analysis [5]. The growing integration of physical activity recognition systems into medical devices has enhanced monitoring by continuously assessing parameters such as heart rate, pulse analysis, and blood pressure [6,7]. While HAR is widely utilized in sports medicine and high-performance physical training, its adoption in certain healthcare domains remains limited. For instance, physical rehabilitation medicine relies heavily on expert interpretation to assess motor function. Moreover, certain neurodegenerative diseases, such as PD and multiple sclerosis, present movement impairments that require early and accurate diagnosis. These conditions’ insidious onset and progressive nature pose significant challenges for timely detection, often resulting in diagnostic uncertainty, particularly in the early stages [8,9].

Although body sensors have been widely recognized as valuable tools for monitoring physical activity, several challenges persist in developing HAR-based diagnostic systems. These include sensor performance variability, the optimal number and placement of sensors, data processing methodologies, and the mathematical models used for data interpretation. Sensor performance can be affected by internal factors, such as hardware limitations or failures, as well as external influences, including environmental conditions like magnetic interference or temperature fluctuations [10]. To mitigate these problems, a robust preprocessing process is crucial for improving data quality and reliability. Additionally, sensor selection and placement must be carefully optimized based on the specific characteristics of the analyzed motion and the target pathology [11,12]. While deep learning techniques have been widely explored in HAR applications, their use in PD diagnosis remains relatively limited [13,14]. Existing models often require significant optimization to improve interpretability, generalizability, and adaptability for broader applications beyond PD detection.

To address these challenges, we propose evaluating a comprehensive HAR process encompassing the entire processing chain, from data collection to the development of standardized classification models. This complete chain constitutes what is referred to as a HAR pipeline. The objectives of this study were as follows: (1) to assess the integration of data from multiple body sensors; (2) to develop and implement a predictive real-time classification model for PD detection; (3) to establish a generic and adaptable HAR pipeline; and (4) to thoroughly test and evaluate system performance. This work builds upon existing HAR approaches, introducing several innovative components. First, we leveraged Motigravity, a novel hypogravity simulation platform, to provide a standardized and clinically relevant context for gait analysis in PD. Second, we integrated wearable inertial sensors in a real-time acquisition setup, highlighting the system’s practical feasibility for future use in clinical settings. Third, we proposed a structured data segmentation strategy, designed to improve dataset balance and segment quality, particularly valuable in data-limited contexts. Finally, we implemented a hybrid CNN-LSTM (Convolutional Neural Networks—Long Short-Term Memory) model, specifically optimized to capture both the spatial and temporal dynamics of Parkinsonian movement.

This study is presented as a technical proof-of-concept model to explore the feasibility and internal consistency of a wearable sensor-based HAR pipeline for the diagnosis of PD. It relies on a small but carefully curated dataset with limited inter-subject variability, and is not intended to support clinical generalization at this stage.

## 2. Materials and Methods

### 2.1. Study Design

This study employed a cross-sectional observational design, based on the analysis of previously collected data from patients diagnosed with PD and healthy volunteers. The patient data were collected during a prior clinical study conducted at the Aragon Parkinson’s Association in Zaragoza, Spain, and approved by the Aragon Research Ethics Committee (CEICA) under registration number C.P.-C.I. PI18/386, in compliance with the Declaration of Helsinki and all relevant ethical guidelines. All participants were thoroughly informed about the study’s objectives, procedures, and potential risks, including muscle fatigue or dizziness related to movement tasks. Written informed consent was obtained from each participant before enrollment. Although the specific data used in the present study had not been previously analyzed in publications, the collection was conducted under the same clinical agreement and with the same ethical approval as the original intervention study.

Eight individuals with PD and one healthy subject participated. Movements were recorded using wearable inertial sensors during controlled walking sessions. Data augmentation was later applied to the healthy recordings to enable comparative analysis.

Although only one healthy individual was included, the dataset was augmented by extracting hundreds of 5 s signal segments from curated video-aligned recordings. The selected segment counts correspond to subsets that yielded the most stable classification results during model evaluation. This approach allowed for the exploration of intra-class variability and model consistency across experimental configurations, while acknowledging the absence of inter-subject variation.

### 2.2. Participants

Participants in this study were recruited as part of a previous clinical project conducted at the Aragon Parkinson’s Association in Zaragoza, Spain, during 2019. Dissemination and recruitment were organized through information sessions held at the association’s premises, where potential candidates were informed about the study objectives, procedures, inclusion criteria, and associated risks. Recruitment was conducted voluntarily among the association’s members.

On the one hand, the eligibility criteria for patients with PD included a confirmed diagnosis of idiopathic PD by a neurologist, the ability to walk independently for short distances without the use of assistive devices, and a stable clinical condition that allowed for participation in motor tasks. Patients with severe cognitive impairment, orthopedic conditions affecting gait, or other neurological diseases were excluded. On the other hand, the healthy subject was recruited separately and selected based on the following eligibility criteria: absence of neurological, musculoskeletal, or balance disorders; no history of movement impairments; and the ability to perform walking tasks safely without assistance.

### 2.3. Equipment

The Motigravity system was used. It consists of a convex platform that integrates multidirectional movements into a monodirectional treadmill. Motigravity (manufactured by Aldebran Srl, Curno, BG, Italy) is an immersive device initially developed for simulating space operations, which has been later adapted for use in physical and medical rehabilitation [15,16,17]. The system integrates a virtual reality headset to provide patients with a fully immersive 360° environment that enhances engagement during therapy sessions [18]. To simulate weightlessness, the device uses a lifting mechanism that supports the patient using a sling, thereby reducing their perceived body weight [19]. This configuration minimizes physical effort and allows patients to perform motor exercises more easily [17].

**Figure 1:**
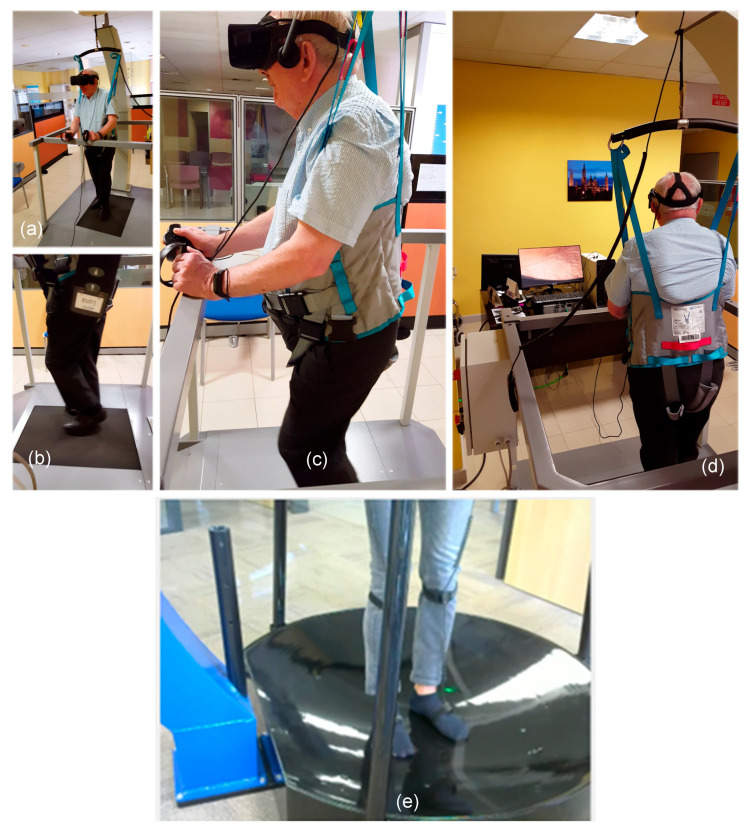
(**a**–**d**) Different points of view of the Motigravity system incorporating a monodirectional treadmill for rehabilitation exercises. The subject is secured with a harness system, effectively simulating a hypogravity environment. This setup enables individuals, including those with PD, to perform controlled movements while reducing gravitational strain and minimizing injury risks. A virtual reality headset is included to improve patient motivation and adherence to therapeutic sessions. (**e**) An illustration of Motigravity with the convex platform used by the healthy subject.

In this study, the healthy volunteer performed exercises on the convex treadmill (Figure 1b), while PD patients used the monodirectional treadmill as part of their movement rehabilitation protocol (Figure 1a–d). Both lifting systems were similar in structure; however, the monodirectional treadmill offered greater safety and accessibility for patients with PD. In contrast, the curved treadmill was better suited for healthy individuals and sports medicine applications.

**Figure 2:**
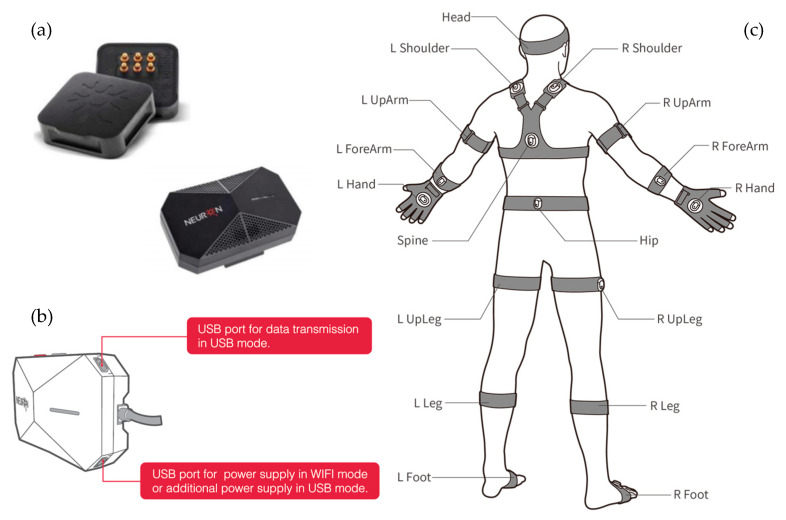
The Perception Neuron motion capture system was used in this study. (**a**) Inertial Measurement Unit (IMU) comprises a 3-axis gyroscope, accelerometer, and magnetometer. (**b**) The data acquisition hub collects real-time motion data from multiple Neuron sensors and transmits it to the computer via USB or a wireless connection. (**c**) Elastic straps secured the sensors in their optimal anatomical positions to ensure accurate and consistent data capture.

The Perception Neuron (PN) 2.0 system (Figure 2) was utilized for motion data acquisition, a motion capture technology widely employed in various fields, including video game development, film, biomechanical research, and sports analysis [20]. The PN 2.0 system consists of several small sensors, called neurons, each containing the following: (1) a 3-axis accelerometer (X, Y, Z) to measure changes in acceleration, albeit influenced by gravity; (2) a 3-axis gyroscope (X, Y, Z) to measure angular velocity, which operates at high frequencies but consumes significant power; and (3) a 3-axis magnetometer (X, Y, Z) to measure magnetic fields, which is highly sensitive to external disturbances. Motion data were visualized and recorded using Axis Neuron software (version 3.8.42.8591), which allows for real-time motion transmission and exports data in two formats: computed values and BVH (BioVision Hierarchy).

### 2.4. Assessment and Data Processing Methods

As mentioned above, gait was measured with the PN 2.0 system (Noitom, Beijing, China). A regular camera recorded the motion sequences, capturing detailed visual data for each activity [21]. Each video sequence was segmented into eight synthetic sequences to isolate the portion of interest [22], focusing specifically on the desired movement. Each segmented sequence was then assigned an appropriate label. Corresponding to each labeled segment, a CSV file containing the sensor data recorded during the activity acquisition was generated. These CSV files represent multivariate time series, with feature vectors extracted from the sensor signals to capture key motion characteristics.

Before any processing steps, each recorded session was manually reviewed to identify and retain clinically relevant intervals based on video alignment and signal quality. Only these validated intervals were passed to the sliding window segmentation process described below.

To ensure consistency across all sensor data, interpolation was applied to standardize the sampling rate to 25 Hz. This step was crucial for aligning signals from multiple sensors and minimizing temporal mismatches. A Savitzky–Golay filter was used to smooth the signals while preserving essential characteristics, such as sharp variations indicative of specific movement patterns. A Butterworth low-pass filter was also applied to remove high-frequency noise and separate body motion components from gravitational effects, particularly in accelerometer data [23,24]. Normalization was performed using a zero-mean and unit-variance transformation to harmonize data across different sensor dimensions, reducing the impact of varying units and measurement ranges. Overall, these preprocessing techniques significantly increased the robustness of subsequent analyses by reducing noise and artefact interference, thereby improving data quality for feature extraction and classification tasks.

Each sensor captures multiple values over time (e.g., a 3D accelerometer records data along the X, Y, and Z axes). To represent these outputs, we used vector notation: $si=(d_{1},\;d_{2},\;d_{3},\;\ldots \;,\;d_{t})$ where i represents the sensor index (1 to k); k is the total number of sensors; and $d_{t}$ is the recorded value at time t. The raw multivariate time series data were transformed into a preprocessed time series to ensure that data processing is standardized and independent of individual patient characteristics. This new representation d refers to one dimension of the processed data, n which is the total number of data dimensions, and t represents the number of samples. This transformation enhances model robustness by applying signal processing techniques that filter out noise and remove artifacts while preserving important motion patterns.

To address the initial class imbalance in our dataset, where only one healthy subject was available compared to eight PD patients, we implemented a minimal data augmentation strategy exclusively on the healthy signals [24,25,26]. This approach aimed to generate a sufficient number of clean segments for training while preserving the integrity of the original data. Among standard augmentation techniques for time-series data (such as Scaling, Jittering, and Time Warping), only Time Warping (TW) was applied in our study [14]. TW introduces smooth, non-linear variations in the temporal dimension of the signal, mimicking natural fluctuations in gait without altering the physiological structure of the movement. This was particularly important given the clinical context of the data. We deliberately did not apply Scaling or Jittering, as these methods introduce variations in signal amplitude or inject random noise, which could compromise the data’s biomechanical interpretability and clinical relevance, especially when applied to sensor signals derived from a single individual. The decision to limit augmentation to TW was made to ensure that the dataset remained as representative and physiologically grounded as possible, while still providing enough healthy segments for practical model training and comparison.

To maximize recognition accuracy and create a sufficient number of training samples, we applied a non-overlapping sliding window segmentation process [27,28,29] to the raw inertial signals. The full-time series *W* was divided into fixed-length segments *w_i_*, each representing a 5 s time window (125 time points). Formally, the segmentation can be described as follows:

W={w0,w1,…,wm−1},wi=(t1,t2) with ∣t2−t1∣=T

where each window *w_i_* is defined by its start and end times in the series, and *T* is the fixed duration of each segment.

Each segment was automatically assigned a label based on the class of the original subject from which it was extracted: either Parkinson’s or Healthy. To ensure data quality, segments that were incomplete or triggered interpolation errors were automatically excluded through error handling routines during the preprocessing stage. No manual filtering was performed after segmentation.

An automated feature selection strategy was implemented to identify the most informative sensor inputs and reduce the model’s dimensionality. The process involved three key steps:

- Sensor contribution analysis: A Random Forest classifier was trained using features from all sensor locations and axes (X, Y, and Z), and feature importance scores were computed for each input [29,30,31].
- Elimination of non-informative inputs: The final model excluded sensors and axes of little to no importance. Specifically, the *Z*-axis of the right ankle sensor and the *X*-axis of the lower back sensor were found to contribute minimally to the CNN-LSTM’s accuracy and were, therefore, removed.
- Sensor subset optimization: Iterative models were constructed using subsets of the remaining sensors and axes to identify the combination that yielded the best classification performance.

Only sensor axes demonstrating consistent and high importance were retained in the final feature set. This refined configuration improved both the model’s interpretability and computational efficiency.

A Convolutional Neural Network—Long Short-Term Memory (CNN-LSTM) model was selected to classify PD movement patterns by capturing both spatial and temporal features [25]. The architecture consisted of the following components:

1. Two convolutional layers with 32 and 64 filters, respectively, using ReLU activation functions to extract local spatial features from the input sequences;
2. A stacked LSTM layer with 128 units to model temporal dependencies across time steps;
3. A fully connected dense layer followed by a SoftMax output layer for binary classification;
4. Dropout (with a rate of 0.5) and batch normalization were applied to prevent overfitting and improve generalization.
5. Hyperparameters were optimized using the Adam optimizer (learning rate = 0.001), with a batch size of 32 during training.

### 2.5. Intervention

**Figure 3:**
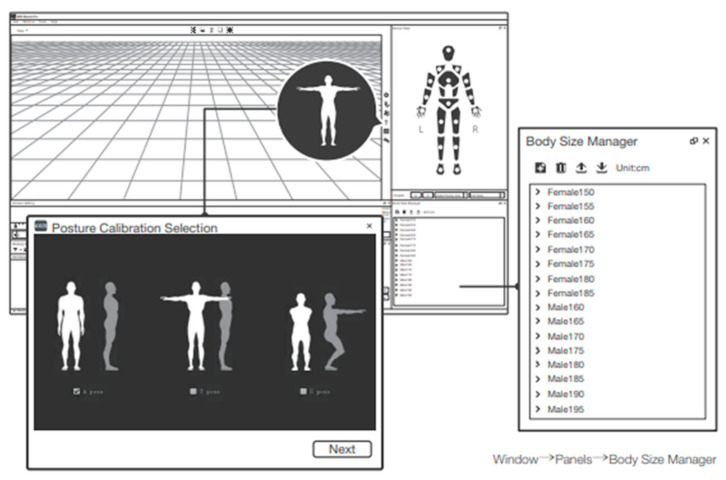
User interface of the Perception Neuron 2 calibration and configuration process. The Posture Calibration Selection window allows users to select among Steady Pose, A-Pose, T-Pose, and S-Pose options to initialize sensor orientation and body alignment. The Body Size Manager panel enables you to choose predefined anthropometric templates based on gender and height, ensuring accurate skeletal scaling during motion capture. These initial configurations are crucial for establishing a standardized reference frame before commencing data acquisition. (Source: https://support.neuronmocap.com, accessed on 24 January 2025).

Before data acquisition, a comprehensive posture calibration procedure was conducted to ensure accurate alignment of the inertial measurement units (IMUs) integrated into the PN 2.0 system (Figure 3). Participants were sequentially instructed to perform a four-step calibration sequence, consisting of a steady pose (standing upright, arms relaxed alongside the body), an A-Pose (arms abducted at approximately 30–45° from the torso with palms facing downward), a T-Pose (arms extended horizontally at 90°, forming a “T” shape with the torso), and an S-Pose (slight knee flexion with arms projected forward at a 45° angle). Each pose was held steadily for several seconds and captured using the Axis Neuron software to minimize initialization errors and standardize the body reference frame across participants.

Following successful calibration, participants were instructed to perform a natural walking task on the Motigravity platform, designed to simulate hypogravity conditions. To preserve ecological validity, they walked at a self-selected, comfortable pace, without specific constraints on cadence, arm swing, or posture. Each session consisted of multiple walking trials. During these tasks, inertial sensor data were recorded simultaneously with synchronized video sequences, allowing for both quantitative kinematic analysis and qualitative review of movement patterns. The total recording duration per participant typically ranged between 3 and 10 min, depending on individual walking speed, endurance, and the number of walking cycles performed. This variability reflects the naturalistic protocol design, in which participants were instructed to walk at a self-selected, comfortable pace without external pacing constraints.

Patients with PD benefited from additional treadmill support and Physical and Medical Rehabilitation assistance provided by the Motigravity system to enhance safety and minimize fall risks. In contrast, the healthy subject performed the task with minimal assistance.

All experiments were conducted in a controlled laboratory setting using a hypogravity treadmill with consistent lighting, surface, and supervision. These conditions do not reflect the variability typically encountered in clinical practice. Thus, no external validation or cross-validation was performed. This work focuses on assessing internal feasibility, and future studies should include inter-subject or external test set evaluations.

### 2.6. Analysis

Data analysis focused on evaluating the training dynamics and classification performance of the CNN-LSTM model through descriptive metrics and graphical representations derived directly from the model outputs. Given the small cohort size and the use of synthetic data for healthy segments, inferential statistics were not deemed appropriate. Instead, the focus was placed on robust training and validation performance across multiple experimental configurations, as well as the reproducibility of the classification results. This process was based on the study by Skolova et al. [32], who comprehensively analyzed the most commonly used performance metrics in classification tasks, including accuracy, recall, F1 score, and the area under the curve (AUC). Their work emphasizes that metric selection should be context-specific, especially in cases of imbalanced data or multi-class environments. It highlights the importance of using multiple complementary metrics to assess model performance robustly [32].

After training, key performance indicators were systematically extracted from the model’s internal history logs, including training loss, validation loss, training accuracy, and validation accuracy. These metrics were plotted across the full number of epochs to assess model convergence, risk of overfitting, and generalization behavior. Loss curves were generated to monitor the decrease in the cross-entropy loss during learning, while accuracy curves illustrated the evolution of correct classification rates over time.

Confusion matrices were computed for each experimental configuration to quantify the classification effectiveness. These matrices detailed the counts of true positives, true negatives, false positives, and false negatives, enabling a transparent view of misclassification patterns if present.

Additionally, Receiver Operating Characteristic (ROC) curves were produced by plotting the true positive rate (sensitivity) against the false positive rate (1-specificity) at various classification thresholds. The AUC metric was calculated to provide a single-value summary of the classifier’s discriminative power.

All analyses and figure generation were conducted using Python 3.8, primarily utilizing the following libraries: TensorFlow for model training history extraction, scikit-learn for confusion matrix computation and plotting ROC curves, and Matplotlib (version 3.0.3) for visualizing all curves and diagrams. Figures were formatted to maintain consistency in axis scaling, color schemes, and annotation styles for publication-quality appearance. No manual post-processing or result alteration was applied to the generated curves.

## 3. Results

This study included eight patients diagnosed with PD and one healthy subject. Our data augmentation technique controlled temporal variations while preserving the underlying kinematic structure of gait, allowing the generation of 20, 107, and 247 synthetic segments for experimental configurations.

The three values used for the healthy group segment counts (20, 107, and 247) were selected empirically after testing several configurations. These values correspond to stable performance, with high classification accuracy and low variance across repeated runs. Smaller datasets led to underfitting and unstable training, while larger sets (>300) introduced redundant or overly similar segments, occasionally degrading generalization due to artificial regularities. Therefore, we retained only the segment sizes that yielded consistent and interpretable results under controlled conditions.

**Figure 4:**
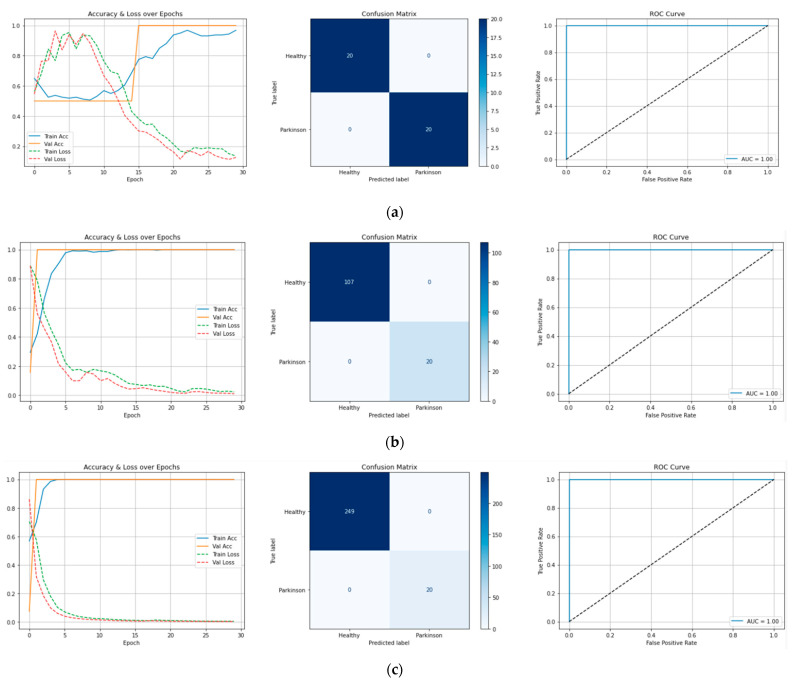
Performance of the CNN-LSTM model across three experimental configurations: (**a**) 20 healthy vs. 20 Parkinson segments—rapid convergence, 100% accuracy, AUC = 1.00; (**b**) 107 healthy vs. 20 Parkinson segments—stable generalization despite imbalance, 100% accuracy, AUC = 1.00; (**c**) 247 healthy vs. 20 Parkinson segments—robust performance under high imbalance, 100% accuracy, AUC = 1.00. Loss curves, accuracy curves, confusion matrices, and ROC curves confirm consistent and perfect classification across all scenarios. The solid blue curve represents the model performance (AUC = 1.00). The black dotted diagonal line represents random classification (chance level, AUC = 0.5).

Based on a hybrid CNN-LSTM architecture, the final model was trained on the refined feature set. Across all segment-based experiments (balanced and imbalanced), the model achieved 100% classification accuracy and an AUC of 1.00, with no observed misclassifications (Figure 4). This suggests excellent separability between Parkinsonian and healthy gait patterns, even with a significant class imbalance. Training and validation curves confirmed stable learning: (1) the loss curves steadily decreased and converged after ~150 epochs; (2) accuracy curves stabilized at 100% for both training and validation; and (3) the confusion matrix revealed perfect classification (no false positives or negatives).

## 4. Discussion

The findings of this study highlight the potential of combining wearable sensor data with deep learning for accurately and robustly classifying movement patterns associated with Parkinson’s disease. Our CNN-LSTM model, trained on segmented inertial data from one healthy subject and eight patients with PD, achieved perfect classification performance (100% accuracy and AUC = 1.00) across all tested configurations, even under increasing class imbalance, being higher than the performance observed in previous PD studies [25]. Despite the limited dataset size, our approach demonstrated consistent and perfect discrimination between PD and healthy gait across multiple experimental settings. This highlights the strength of the proposed pipeline and the marked spatiotemporal differences between the two groups. As such, this study provides strong internal validation for the methodology and offers valuable insight into the feasibility of implementing reliable, sensor-based diagnostic tools for PD.

Time Warping (TW) was applied exclusively to the healthy subject’s segments to expand the training dataset synthetically, compensating for the limited number of healthy recordings. This augmentation technique preserved the underlying biomechanical structure of gait while introducing realistic variability [33]. The model maintained its performance across increasingly imbalanced conditions (20:20, 107:20, 247:20). The application of data augmentation and video-based data mining techniques in individuals with PD has already been demonstrated, particularly for the automated detection of hypomimia [34]. These approaches have effectively addressed the challenges posed by small and unbalanced datasets, thus improving model performance.

The hybrid CNN-LSTM architecture was chosen to leverage the complementary strengths of convolutional and recurrent layers [35]. The CNN component effectively extracted spatial features from motion signals, such as local gait patterns and step dynamics. In contrast, the LSTM component modeled temporal dependencies and sequencing, which is crucial for detecting characteristic irregularities in PD movements. This combination proved particularly effective in distinguishing between healthy and pathological movement patterns. Moreover, the model’s balance between accuracy and computational efficiency makes it highly suitable for real-time applications.

Preprocessing played a critical role in ensuring the reliability of the input data in our study. Raw signals were standardized to a sampling frequency of 25 Hz and filtered using a combination of Savitzky–Golay and Butterworth filters, a strategy also highlighted by Celik et al. [23] to improve signal quality in neurological populations. The subsequent feature selection process, based on a Random Forest algorithm, enabled the exclusion of low-informative axes, focusing the model on the most discriminative biomechanical dimensions, particularly those linked to gait and balance control. This approach aligns with the findings of Davidashvilly et al. [25], who demonstrated that targeted feature optimization can enhance model generalization, especially in datasets with limited subject variability.

Notably, by carefully selecting relevant features and filtering noise, we achieved high classification performance without relying on excessively deep or complex network architectures, an issue previously discussed by authors [25] in the context of preventing overfitting in PD activity recognition. Furthermore, our minimal augmentation strategy contrasts with more aggressive approaches, such as the use of Generative Adversarial Networks (GANs) proposed by Lupion et al. [26], indicating that even limited augmentation, combined with rigorous preprocessing, can yield excellent results when movement patterns are sufficiently distinct between classes.

These findings support the critical importance of preprocessing and feature engineering as foundational steps in HAR pipelines targeting clinical populations, particularly when working with limited datasets. Before segmentation, raw recordings were manually reviewed to identify and retain only high-quality intervals, based on clinical relevance, video alignment, and signal integrity. The segmentation itself was then fully automated using a sliding window approach. No algorithmic quality filtering was applied after segmentation; instead, segment quality was ensured by careful manual review before automated processing. Significantly, the classification was not based on a single continuous signal but on hundreds of independent 5 s segments, which were extracted and filtered for quality, providing sufficient variability within the healthy class. Moreover, the spatiotemporal differences between Parkinsonian and healthy gait patterns in this dataset were highly distinctive, facilitating perfect separation under these controlled experimental conditions. The 100% classification accuracy was consistently replicated across the three experimental configurations, reinforcing the internal validity of the results. Nevertheless, we acknowledge that generalization to unseen healthy individuals has not yet been demonstrated and should be addressed in future studies involving a larger, more diverse cohort.

Given the model’s ability to detect subtle abnormalities in movement dynamics, it could serve as a tool for early detection of PD, enabling timely clinical intervention and more effective rehabilitation strategies, a direction increasingly supported by recent advances in remote sensor-based PD screening [36]. Coupling the AI-driven classification system with the Motigravity platform could enhance personalized therapy through real-time feedback and adaptive training programs [37].

Nonetheless, some limitations must be acknowledged. Foremost among these is the limited dataset size, mainly including only a single healthy control subject. Despite the internal consistency of results, the use of repeated segments derived from a single healthy subject may have introduced overfitting. Although segmentation and data augmentation techniques successfully introduced considerable intra-class variability, they cannot substitute for the inter-subject diversity essential to robust model generalization. The risk of memorizing subject-specific movement patterns must therefore be acknowledged as a major limitation, and the model’s ability to perform reliably on unseen individuals remains unverified. In addition, confounding variables such as age, gender and clinical stage, factors that can significantly influence gait patterns, were not taken into account, as well as scores from standardized clinical rating scales, such as the Unified Parkinson’s Disease Rating Scale (UPDRS), were not used to correlate movement patterns with disease severity, limiting the clinical interpretability of the findings. Furthermore, the manual selection of artifact-free sequences before segmentation may still introduce selection bias, even when following a standardized procedure. Earlier iterations of this study occasionally exhibited misclassifications, particularly in mild PD cases with variable motor presentations; however, such errors were not observed in the current analysis. This improvement is attributed to refined preprocessing, feature selection, and architecture tuning.

Overall, our findings demonstrate the effectiveness and robustness of the CNN-LSTM model in accurately classifying Parkinsonian gait based on data from wearable inertial sensors. However, future research should consolidate the clinical relevance of this model by addressing several essential directions. First, validating the model across larger, heterogeneous cohorts is imperative, ensuring its generalizability across diverse populations, disease severities, and phenotypic variations, particularly within the broader spectrum of neurodegenerative disorders. Second, future work should focus on improving segmentation to capture subtle motor abnormalities, which is especially critical for early stages or atypical presentations of PD. Although the model achieved perfect accuracy in this study, its robustness in more diverse real-world scenarios remains to be demonstrated. Real-world implementation in rehabilitation centers and hospital settings should be prioritized to assess the model’s integration within clinical workflows, evaluate its practical usability by healthcare professionals, and identify potential technical or organizational barriers to large-scale deployment. Such steps will be key to translating this proof of concept into a viable tool for personalized diagnostics and AI-assisted rehabilitation.

## 5. Conclusions

This study presents a fully integrated HAR pipeline using CNN-LSTM architectures and inertial sensor data for accurate real-time PD gait classification. The model achieved perfect classification performance (100% accuracy, AUC = 1.00), even under significant class imbalance, thanks to optimized preprocessing, feature selection, and a segmentation strategy. These results highlight the potential of hybrid deep learning approaches to distinguish between pathological and healthy motor patterns with high reliability. The results support the clinical feasibility of implementing such models for early PD detection and individualized rehabilitation strategies, especially when integrated with immersive platforms such as Motigravity. Despite promising results, further validation with larger and more diverse cohorts is required to confirm generalizability. This work lays the foundation for future AI-based diagnostic systems that continuously and objectively monitor motor function in clinical and home settings.

## Data Availability

The data presented in this study are available on request from the corresponding author.
